# M1-derived extracellular vesicles enhance photodynamic therapy and promote immunological memory in preclinical models of colon cancer

**DOI:** 10.1186/s12951-022-01448-z

**Published:** 2022-06-03

**Authors:** Ruben V. Huis in ‘t Veld, Pablo Lara, Martine J. Jager, Roman I. Koning, Ferry Ossendorp, Luis J. Cruz

**Affiliations:** 1 Department of Radiology, Leiden University Medical Centre (LUMC), Room C2-187h, Albinusdreef 2, 2333 ZA Leiden, The Netherlands; 2 Department of Ophthalmology, Leiden University Medical Centre (LUMC), Leiden, The Netherlands; 3 Department of Immunology, Leiden University Medical Centre (LUMC), Leiden, The Netherlands; 4 Department of Cell and Chemical Biology, Section Electron Microscopy, Leiden University Medical Centre (LUMC), Leiden, The Netherlands

**Keywords:** Photodynamic therapy, Extracellular vesicles, Exosomes, Cancer, Tumor microenvironment, Immune modulation, Theranostics, Pharmacology, Delivery, Macrophages

## Abstract

Extracellular vesicles (EVs) are promising drug carriers of photosensitizers for photodynamic therapy (PDT) in cancer treatment, due to their ability to circulate in blood and enter cells efficiently. The therapeutic potential of EVs has been suggested to depend on the type and physiological state of their cell of origin. However, the effects of deriving EVs from various cells in different physiological states on their antitumor capacity are rarely evaluated. In the present study, we compared the antitumor efficacy of EV-mediated PDT by incorporating the photosensitizer Zinc Phthalocyanine (ZnPc) into EVs from multiple cells sources. ZnPc was incorporated by a direct incubation strategy into EVs derived from immune cells (M1-like macrophages and M2-like macrophages), cancer cells (B16F10 melanoma cancer cells) and external sources (milk). Our data show that all EVs are suitable carriers for ZnPc and enable efficient PDT in vitro in co-culture models and in vivo. We observed that EV-mediated PDT initiates immunogenic cell death through the release and exposure of damage associated molecular patterns (DAMPs) on cancer cells, which subsequently induced dendritic cell (DC) maturation. Importantly, of all ZnPc-EVs tested, in absence of light only M1-ZnPc displayed toxicity to MC38, but not to DC, in monoculture and in co-culture, indicating specificity for cancer over immune cells. In MC38 tumor-bearing mice, only M1-ZnPc induced a tumor growth delay compared to control in absence of light. Interestingly, M1- but not M2-mediated PDT, induced complete responses against MC38 tumors in murine models (100% versus 38% of cases, respectively), with survival of all animals up to at least 60 days post inoculation. Finally, we show that all cured animals are protected from a rechallenge with MC38 cells, suggesting the induction of immunological memory after EV-mediated PDT. Together, our data show the importance of the cell type from which the EVs are obtained and highlight the impact of the immunological state of these cells on the antitumor efficacy of EV-mediated PDT.

**Graphical Abstract:**

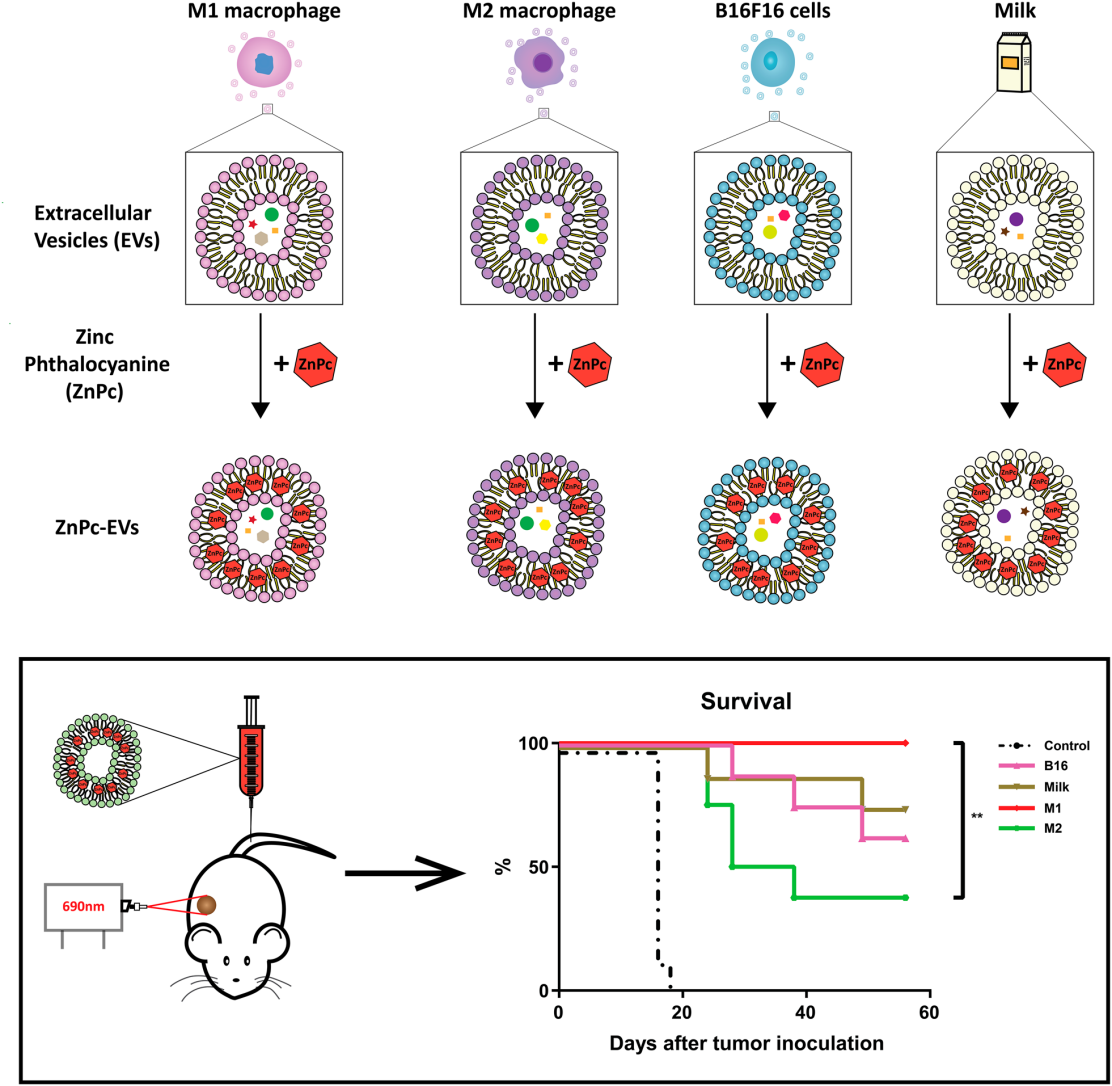

**Supplementary Information:**

The online version contains supplementary material available at 10.1186/s12951-022-01448-z.

## Introduction

In cancer treatment, photosensitizers (PS) are used as light-transferring molecules for photodynamic therapy (PDT) that generate reactive oxygen species (ROS) and facilitate damage to cells and structures in the tumor area. A longstanding limitation of many PS, e.g. Zinc Phthalocyanine (ZnPc), is their unfavorable distribution upon intravenous administration, after which a poor accumulation in the tumor and off-target distribution are often observed (e.g. the skin), resulting in prolonged periods of skin hypersensitivity. Incorporation of PS in nano-sized carrier systems may overcome such limitations, allowing control over the biodistribution of the encapsulated agents defined by the characteristics of the carrier. Recently, small extracellular vesicles (EVs) have been identified as excellent (nano) carrier systems for antineoplastic agents due to their favorable pharmacokinetic properties and ability to easily enter their target cells [1–3]. EVs are cell-derived vesicles with sizes ranging between 30 and 2000 nm that are involved in cell–cell communication and immunological processes e.g. antigen presentation (reviewed in [4]). They consist of a lipid-bilayer with various membrane proteins and have been shown to carry numerous proteins, lipids and genetic information including DNA, mRNA, lncRNA and microRNA [5–9]. The composition of the lipid-bilayer and the specific content of the EVs are dependent on the type as well as the physiological and immunological state of their cell of origin and can therefore vary [4]. Interestingly, EVs can also have unique properties depending on their origin such as oral absorption (milk-EV), antitumoral properties (M1-like EVs), anti-inflammatory properties (grape EVs) or tumor targeting (B16F10-EVs) [10, 11]. EVs can also present antiphagocytic markers increasing their time circulating in blood [12] and have been loaded with multiple therapeutic compounds such as microRNAs [13, 14], doxorubicin [15] and paclitaxel [16], which classify them as highly suitable and versatile carriers for different types of drugs.

The potential of EVs as immunomodulators has been investigated in preclinical studies and clinical trials [17–23]. However, the clinical efficacy of EVs remained insufficient to result in the introduction of EVs as standard of care. This may be explained in part by the notion that the role of EVs as a standalone treatment of cancer has been controversial and difficult to interpret due to the plethora of EVs natively present in the tumor, as well as the ability of cancer cells to develop drug resistance and escape immune surveillance. In line with this, the use of EVs has resulted in both tumor progression [25, 26] and regression [26–28], varying between studies that employed EVs derived from different sources. In a recent preclinical study, however, EVs derived from M1-polarized macrophages containing paclitaxel induced a tumor growth delay in 4T1 tumor-bearing mice [24]. Notably, the M1-derived EVs alone also inhibited tumor growth to which the addition of paclitaxel did not result in a significant benefit in survival. These results showed the potential of M1-derived EVs to inhibit tumor growth, even in absence of pre-loading with drugs.

We have previously shown that B16F10-derived EVs are excellent carriers of the potent, but poorly soluble PS ZnPc [29]. The EVs containing ZnPc (ZnPc-EVs) were easily prepared and displayed favorable characteristics for cancer treatment in vitro. Furthermore, the ZnPc-EVs were shown to distribute to Murine Colon 38 (MC38) tumors after intravenous administration over time. Photodynamic therapy with the ZnPc-EVs strongly inhibited MC38 tumor growth in murine models, but the long-term effects and survival benefit were not explored. In the present study, we aimed to improve our treatment and compare the antitumor efficacy of EVs derived from various sources in different immunological states. Due to previous reports on the antitumor efficacy of M1-derived EVs [24], we hypothesized whether they would enhance ZnPc-PDT treatment compared to the (B16F10) cancer cell-derived EVs. Moreover, we investigated whether the immunological state of the cell influences the efficacy of the EVs, by comparing ZnPc-containing M1-derived (proinflammatory) EVs with M2-derived (immunosuppressive) EVs. In addition, we investigated whether non-cancer or immune cell-derived EVs would display differences in antitumor efficacy against MC38 tumors in murine models, and have chosen bovine milk-derived EVs due to their availability and ease of upscaling. We show that EVs of various origins, namely B16F10 cells, M1- and M2 polarized macrophages and bovine milk, are excellent carriers of poorly soluble PS and were able to induce efficient immunogenic cell death in vitro, and high antitumor efficacy in vivo. Our data indicates that the cell type and the immunological state from which the EVs are derived have a strong effect on their therapeutic efficacy in the context of PDT which could be exploited for the generation of more effective and personalized treatments.

## Results

### Preparation and characterization of ZnPc-EVs

**Fig. 1:**
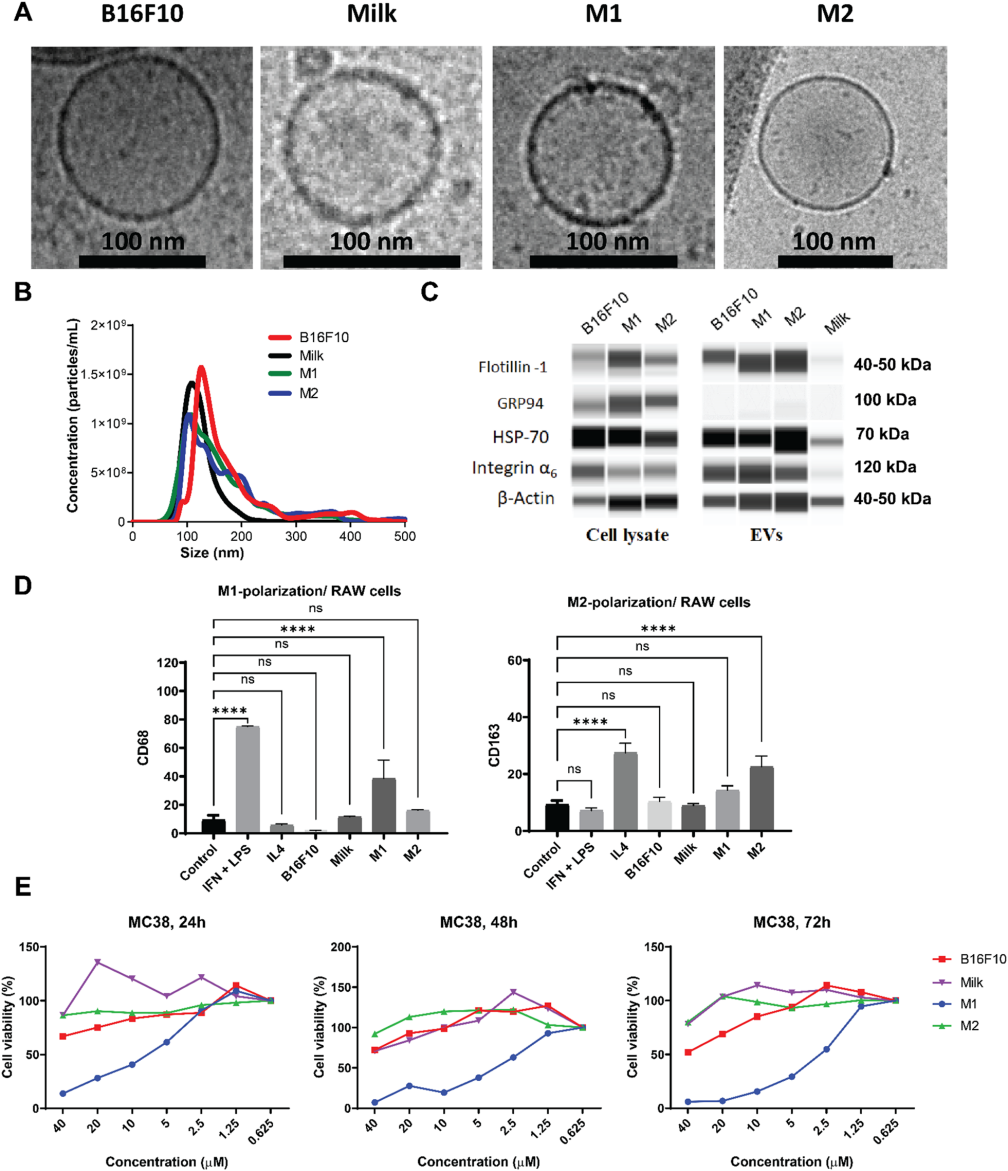
Preparation and characterization of ZnPc-EVs. Representative **a** Cryo-TEM micrograph, **b** size distribution by NTA, **c** western blot analysis of EV markers and **d** M1- polarized RAW 264.7 cells were investigated for CD68 expression (left panel) and M2-polarized RAW 264.7 cells were investigated for CD163 expression (right panel) by flow cytometry after incubation with the ZnPc-EVs, IFN-y and LPS, or IL-4 for 24h. **e** Viability of MC38 cells after incubation for 24, 48 or 72 h various concentration of the ZnPc-EVs. (scale bars are 10 nm.****P < 0.0001; mean ± SEM; n = 3)

EVs were obtained from defatted bovine milk, from the culture media of B16F10 cancer cells, as well as from M1- and M2-polarized RAW264.7 cells. Polarization of naïve RAW264.7 cells to an M1-like phenotype was performed by incubation with LPS and IFN-γ for 24 h, while polarization of naïve RAW264.7 cells to an M2-like phenotype was achieved by incubation with IL-4 for 24 h as described in the methods section. Isolation of EVs from milk and culture media was performed using a formerly established protocol [11], and incorporation of ZnPc was achieved as described previously [29]. Briefly, EVs were incubated with 9 mM ZnPc using an EV/DMSO proportion of 9:1 to obtain the highest concentration of ZnPc without significantly affecting the vesicular distribution of the EVs. The resulting EVs, B16-ZnPc, Milk-ZnPc, M1-ZnPc and M2-ZnPc, displayed a typical spherical shape on (cryo-) transmission electron microscopy (TEM) images (Fig. 1a and Additional file 1: Fig. S1a). The size distributions of the EVs were relatively homogenous, with the majority of EVs ranging from approximately 100–200 nm (Fig. 1b). The presence of cell surface markers Flotillin-1 and integrin α_6_ was observed on all cell culture-derived EVs, but poorly observed in milk samples (Fig. 1c). The vesicle marker heat-shock protein 70 and cytoskeletal marker β-actin were found on all cells and EVs (Fig. 1c), whereas the endothelial marker GRP94 (negative control) was only detected on cells and not on EVs (Fig. 1c). These data correspond to the expected shape, size distribution and protein profiles of EVs, indicating that our preparation was enriched in small EVs of < 200 nm. The ability of the EVs to polarize macrophages was investigated after incubation with RAW264.7 cells for 24 h. Only the M1-ZnPc induced a significant change in the M1-like phenotype marker CD68 compared to control, whereas the EVs M2-ZnPc, B16-ZnPc and Milk-ZnPc did not (Fig. 1d). Similarly, only the M2-ZnPc induced a significant upregulation of M2-phenotype marker CD163 (Fig. 1d) compared to control, indicating the potential of M1-ZnPc to induce M1-polarization and of M2-ZnPc to induce M2-polarization in RAW264.7 cells. The direct effect of the ZnPc-containing EVs on the viability of MC38 cancer cells in absence of light for PDT was investigated by MTS, showing that M1-ZnPc reduced MC38 viability in a concentration dependent manner (Fig. 1e) up to at least 72 h, whereas B16-ZnPc, Milk-ZnPc and M2-ZnPc only slightly affected viability at high concentrations. These results indicate that M1-EVs can induce macrophage polarization towards a pro-inflammatory M1-like phenotype and strongly reduce the viability of MC38 cells in a concentration-dependent manner, while M2-EVs can induce an anti-inflammatory M2-like phenotype in macrophages.

### ZnPc-EVs show a preferential association with cancer cells versus immune cells

**Fig. 2:**
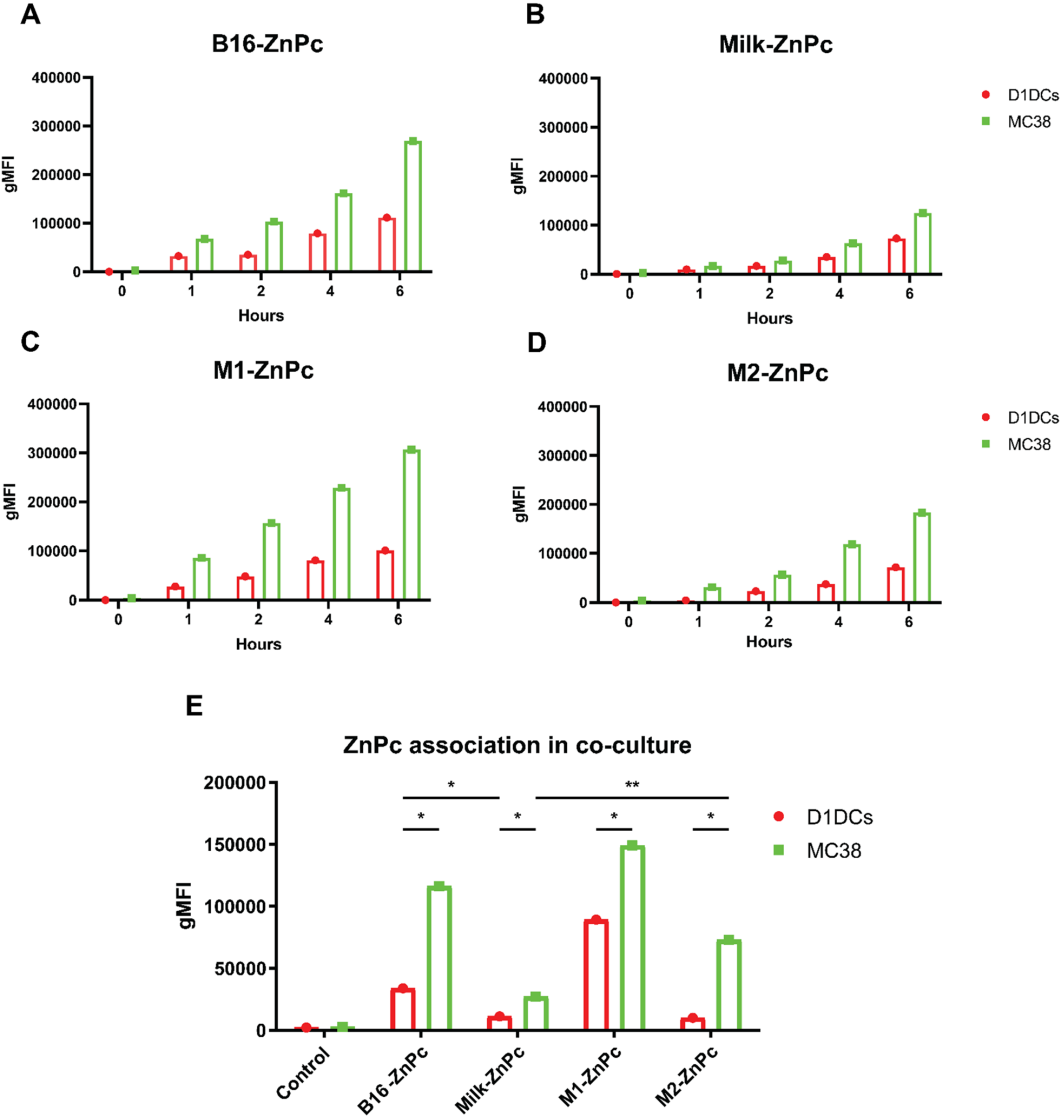
Association of ZnPc-EVs with cancer cells and dendritic cells. MC38 or D1DCs were incubated with **a** B16-ZnPc, **b** Milk-ZnPc, **c** M1-ZnPc and **d** M2-ZnPc for 0–6 h. Fluorescence of ZnPc was measured in the cells by flow cytometry. **e** Equal numbers of a co-culture of MC38CFP and D1DCs were incubated with the ZnPc-EVs for 4 h, after which the fluorescence of ZnPc was measured by flow cytometry. Separation of cell lines was based on gating for CFP+ (cancer cells) or CD11c+ (D1DCs). Statistical significance was determined by Students *t*-test (**P<0.01, *P<0.05; mean±SEM; n=3)

EVs are equipped with natural targeting ligands (e.g., integrins) which can vary depending on the cell source and can have an important impact in their uptake towards specific cells. To test the association in cancer and immune cells, the ZnPc-EVs were incubated with MC38 or D1DCs after which the fluorescence of ZnPc was measured by fluorescence microscopy (Additional file 1: Fig. S1b) and flow cytometry over time (Fig. 2a–d). All ZnPc-EVs induced a measure of association with most of the cells (Additional file 1: Fig. S1b) by fluorescence microscopy. Moreover, when considering percentages of ZnPc^+^ cells by flow cytometry (Additional file 1: Fig. S2a–d), B16-ZnPc and M1-ZnPc were quickly associated with nearly all cells but Milk-ZnPc and M2-ZnPc displayed an increase in the percentage of ZnPc^+^ cells over time. However, B16-ZnPc and M1-ZnPc were most efficiently associated with both MC38 cells and D1DCs when considering the geometric mean fluorescent intensity (gMFI), whereby the gMFI in MC38 cells was consistently enhanced compared to D1DCs (Fig. 2a and c). The Milk-ZnPc and M2-ZnPc displayed a lower association in both MC38 cells and D1DCs, increasing in fluorescent signal over time (Fig. 1b, d). Similar to B16-ZnPc and M1-ZnPc, both Milk-ZnPc and M2-ZnPc displayed a stronger association with MC38 cells versus D1DCs (Fig. 2a–d) which could be related to the enhanced metabolism of cancer cells. As the tumor microenvironment consists of several types of cells interacting closely including cancer and immune cells, we investigated the association of the ZnPc-EVs in a co-culture of MC38 cells and D1DCs. Separation of cells in this co-culture by flow cytometry was based on positivity for CFP (cancer cells) or CD11c (D1DCs) after live gating. In absence of ZnPc (control), as expected, incubation with EVs induced no fluorescence in either MC38 or D1DCs (Fig. 2e). After 4 h of incubation, nearly all cells in co-culture were positive for ZnPc for B16-ZnPc and M1-ZnPc (Additional file 1: Fig. S2e), while incubation with Milk-ZnPc and M2-ZnPc resulted in slightly lower percentages of cells positive for ZnPc at this timepoint (Additional file 1: Fig. S2e). Moreover, we noticed that B16-ZnPc and M2-ZnPc had a strong preference for MC38 over D1DCs (Fig. 2e), with a 3.4-fold increase for B16-ZnPc-EVs and a 7.2-fold increase for M2-ZnPc. Again, M1-ZnPc displayed the strongest fluorescence in all cells tested, but a smaller difference between D1DCs and MC38 cells, with an increased association of approximately 1.7-fold in MC38 versus D1DCs. Milk-ZnPc induced the lowest fluorescence in MC38 cells, with a 2.4-fold lower signal in D1DCs (Fig. 2e), while M2-ZnPc displayed the lowest fluorescence in D1DCs of all EVs tested (Fig. 2e). Together, these results show that M1-ZnPc, M2-ZnPc and B16-ZnPc are efficiently associated with cancer cells, while showing a reduced association with immune cells and that the ZnPc-EVs are display a preferential association with cancer cells over D1DCs in co-culture. The Milk-ZnPc show a similar trend as the other ZnPc-EVs, but display association with significantly reduced efficiency in cancer cells versus all other EVs. The uptake by D1DCs of Milk-ZnPc and M2-ZnPc showed no difference and was significantly lower than the association of B16-ZnPc (Fig. 2e).

### M1-ZnPc preferentially kills cancer cells over immune cells in the dark, while photodynamic therapy efficiently kills all cancer cells

**Fig. 3:**
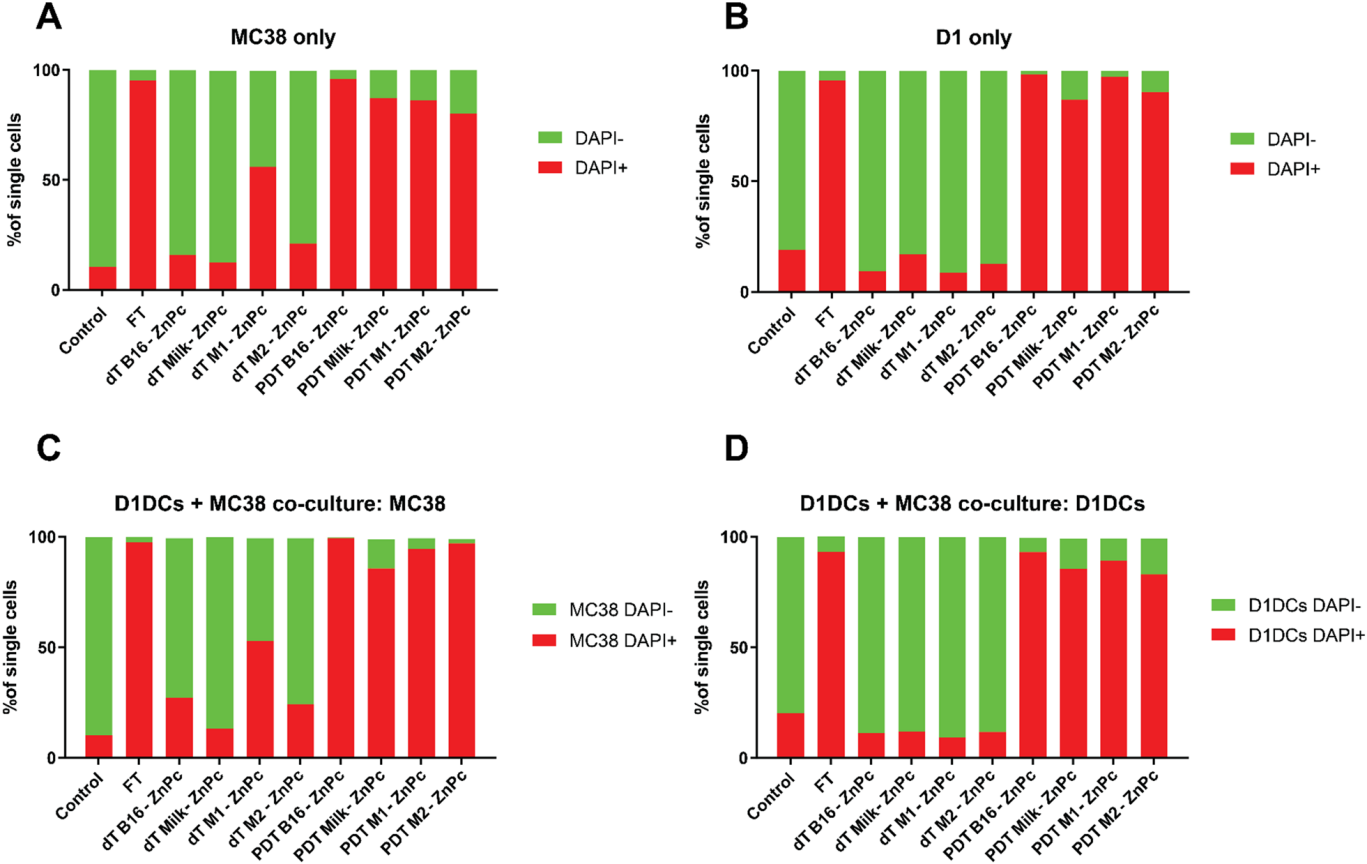
ZnPc-M1 is toxic to cancer cells, but not to D1DCs in absence of light. **a** MC38 or **b** D1DCs were incubated with the ZnPc-EVs for 4 h and left in the dark (dark toxicity, dT), treated with 690 nm at 200 mW/cm^2^ for 25 J/cm^2^ or subjected to three cycles of freeze-thawing at − 20 °C (FT). At 18 h after treatment, the cells were stained for viability marker DAPI and analyzed by flow cytometry. A co-culture of MC38CFP and D1DCs was subjected to the same treatment in addition to antibody staining of CD11c, showing the viability of **c** MC38CFP or **d** D1DCs. Separation of cell lines was based on gating for CFP+ (cancer cells) or CD11c+ (D1DCs). (***P<0.001; **P<0.01; mean±SEM; n=3)

The ability of the ZnPc-EVs to induce cytotoxocity in MC38 cells and D1DCs in the dark (dark toxicity) and after illumination (PDT) was investigated. To this end, Cyan Fluorescent Protein (CFP)-expressing MC38 cells (MC38CFP) were incubated with the ZnPc-EVs for 4 h, washed and subsequently illuminated with 690 nm light at 200 mW/cm^2^ for 25 J/cm^2^. The cells were then incubated for 18 h, stained with death marker DAPI and analyzed by flow cytometry. For MC38CFP, the dark toxicity of B16-ZnPc, M2-ZnPc and Milk-ZnPc was comparable to untreated (control) cells (Fig. 3a). However, the M1-ZnPc induced toxicity in approximately 50% of MC38CFP in absence of light (Fig. 3a). Photodynamic therapy with all ZnPc-EVs tested induced a near-complete cytotoxicity in MC38CFP cells at levels comparable to three cycles of freeze-thawing (FT) at − 20 °C (Fig. 3a). As in MC38CFP cells, the dark toxicity in D1DCs cells exposed to B16-ZnPc, M2-ZnPc and Milk-ZnPc in D1DCs was comparable to control (Fig. 3b), while PDT with all ZnPc-EVs tested induced a near-complete cytotoxicity. Of note, the dark toxicity of M1-ZnPc in D1DCs was comparable to control cells (Fig. 3b), indicating that M1-ZnPc in absence of light is toxic to cancer cells, but not immune cells. A similar trend in cytotoxicity was observed in a co-culture of MC38CFP cells and D1DCs, where the dark toxicity of B16-ZnPc and M2-ZnPc in MC38CFP were slightly increased compared to monoculture (Fig. 3c). However, the dark toxicity of all ZnPc-EVs was similar to control in D1DCs in co-culture (Fig. 3d). Again, PDT with all ZnPc-EVs induced strong cytotoxicity in both MC38CFP cells and D1DCs in co-culture. Importantly, the dark toxicity of M1-ZnPc in co-culture was also increased up to approximately 50% for MC38 (Fig. 3c), but not D1DCs (Fig. 3d). These results indicate that PDT with all ZnPc-EVs efficiently kills tumor and immune cells, but that in absence of light, only M1-ZnPc preferentially induces cytotoxicity in cancer cells over immune cells.

### Photodynamic therapy with ZnPc-EVs induces immunogenic cell death and dendritic cell maturation

**Fig. 4:**
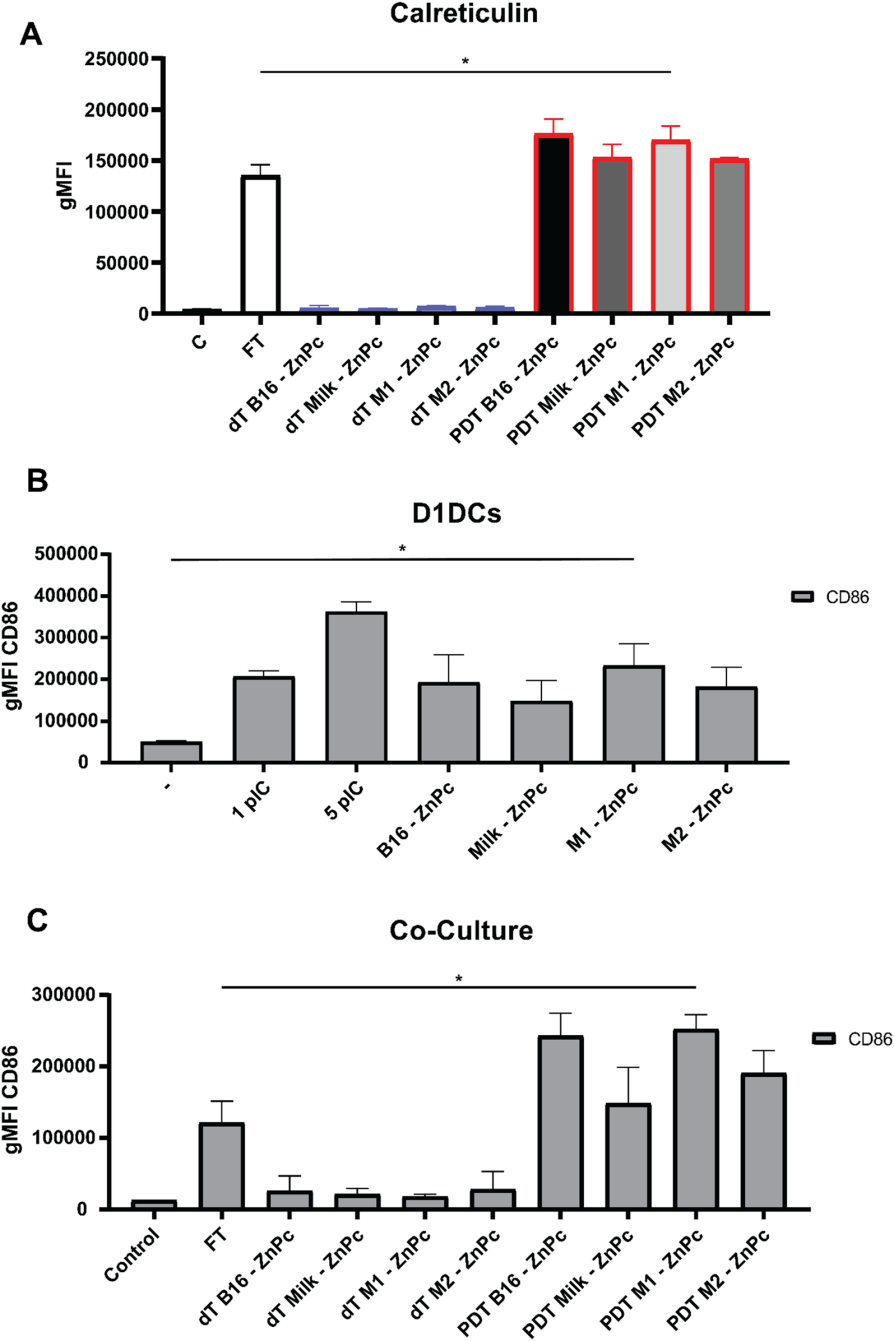
ZnPc-EV-mediated PDT induces calreticulin exposure and dendritic cell maturation. MC38 cells were incubated with the ZnPc-EVs for 4 h and left in the dark (dark toxicity, dT), treated with 690 nm light at 200 mW/cm^2^ for 25 J/cm^2^ or subjected to three cycles of freeze-thawing at – 20 °C (FT). After 18 h the cells were stained for viability marker DAPI and DAMP calreticulin (CRT) and analyzed by flow cytometry after live-gating. **b** D1DCs were treated with 1 or 5 ng/mL poly I:C, or the ZnPc-EVs for 24 h. Cells were then collected, stained with anti-CD86-FITC antibody and measured by flow cytometry. **c** A co-culture of MC38CFP and D1DCs was subjected to the same treatment as in **a**, after which the cells were stained with DAPI, anti-CD86-FITC and anti-CD11c-PE. Separation of cell lines was based on gating for CFP+ (cancer cells) or CD11c+ (D1DCs) events after live-gating. Statistical analysis was determined by Students *t*-test (*P<0.05; mean±SEM; n=3)

Photodynamic therapy has been reported to induce immunogenic cell death (ICD) that leads to the induction of antitumor immune responses [30, 31]. During PDT-induced ICD, damage-associated molecular patterns (DAMPs) are exposed and/or released that initiate an inflammatory response mediated by innate immune cells [30]. Calreticulin (CRT) [31, 32] and high-mobility group box 1 (HMGB-1) [33] have been shown to be important DAMPs in PDT-induced ICD. We investigated whether the ZnPc-EVs induce ICD through exposure of CRT and release of HMGB-1. To this end, MC38 cells were incubated with ZnPc-EVs for 4 h and treated with PDT as described. At 18 h after treatment, CRT was stained after which the cells were analyzed by flow cytometry. In absence of light, the levels of CRT were not altered after incubation with all ZnPc-EVs (Fig. 4a). However, PDT strongly increased CRT exposure at levels comparable to or higher than FT for all ZnPc-EVs (Fig. 4a). There was a slight elevation in the levels of HMGB-1 after PDT with all ZnPc-EVs compared to FT in the supernatant after PDT treatment as described (Additional file 1: Fig. S3). However, HMGB-1 release from MC38 cells in general was reported to be relatively low compared to other cancer cell lines [34]. This notion is underlined by the marginal increase of HMGB-1 release observed for the positive control FT in this study (Additional file 1: Fig. S3). Subsequently, we investigated whether the PDT-induced ICD as exemplified by DAMP exposure could also facilitate maturation of innate immune cells, thereby potentially initiating an inflammatory response. Expression levels of the maturation marker CD86 in D1DCs were comparable to 1 ng/mL of the toll-like receptor ligand-3 (TLR3) poly I:C after 24 h incubation with all ZnPc-EVs (Fig. 4b). In a co-culture of D1DCs with MC38CFP for 24 h (control, Fig. 4c), the expression of CD86 is downregulated to levels below baseline (-, Fig. 4b), showing the ability of MC38CFP to suppress D1DC maturation (Fig. 4c). Pre-treatment of MC38CFP with the ZnPc-EV for 4 h before incubation with D1DCs, slightly increased CD86 expression levels (Fig. 4c). However, incubation of D1DCs with PDT-treated MC38CFP using the different ZnPc-EVs increased CD86 expression to levels higher than D1DCs incubated with FT treated MC38CFP, of which only M1-ZnPc was significantly inreased compared to FT (Fig. 4c). These results are in line with the observed exposure and/or release of DAMPs after PDT-treatment with the ZnPc-EV and show the ability of the ZnPc-EV-mediated PDT treatment to induce ICD.

### Distribution of ZnPc-EVs to tumors in murine models

**Fig. 5:**
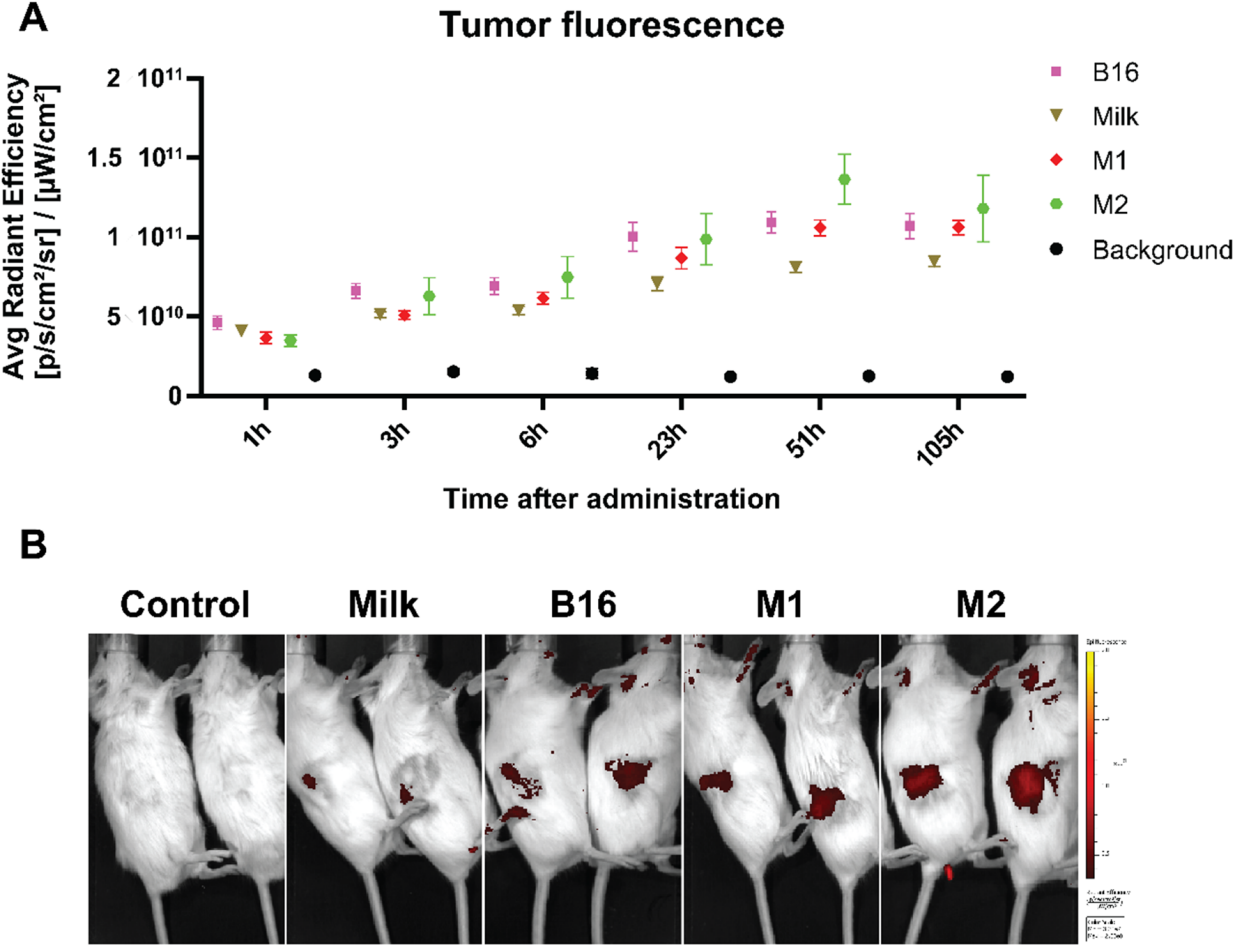
ZnPc-EVs accumulate in tumors following intravenous administration. C57BL/6-albino mice were inoculated with 0.5 × 10^6^ MC38 cells in the right flank. At 7 days after inoculation, when tumors were established (~ 125 mm^3^), the mice were intravenously injected with the ZnPc-EVs in the tail vein. **a** The presence of ZnPc in the tumors was measured over time by fluorescence spectrometry using the IVIS fluorescence spectrometer. **b** Representative IVIS images of the mice with a fluorescence overlay of the 700 nm channel at 51 h after administration are shown. (mean±SEM; n=4)

To follow up on the in vitro cytotoxicity and ability of the ZnPc-EVs to induce DAMP exposure and ICD, we investigated their distribution in MC38 tumor-bearing mice. To this end, C57BL/6 albino mice were subcutaneously inoculated with 0.5 × 10^6^ MC38 cells in the right flank. When the tumors became established (~ 125mm^3^), the ZnPc-EVs were administered intravenously into the tail vein after which fluorescence in the tumors was followed over time. Tumor fluorescence increased over time for all ZnPc-EVs, with a signal peak at ~ 51 h after administration (Fig. 5a). The initial fluorescence of M2-ZnPc was the lowest of all groups at 1 h post administration, increasing up to the highest level around 51 h, but also displaying the highest spread. A similar pattern was observed for B16-ZnPc and M2-ZnPc, with a large increase and relatively high spread between tumors. Both M1-ZnPc and Milk-ZnPc increased steadily over time, of which M1-ZnPc consistantly showed a higher fluorescence in the tumor, with a relatively low spread. Similar to our observations of association with cancer cells in vitro (Fig. 2), Milk-ZnPc displayed the lowest fluorescence in the tumor of all EVs at 6 h post administration (Fig. 5a). However, at the same time point B16-ZnPc and M2-ZnPc displayed slightly higher tumor fluorescence than M1-ZnPc, in contrast to cancer cells in vitro where M1-ZnPc induced the highest fluorescence of all EVs tested (Fig. 2). This observation underlines the difference between in vitro association and distribution in vivo after systemic administration, where multiple additional interactions with the administered molecules are possible compared to in vitro. Together, these results show that all ZnPc-EV accumulate in the tumors over time, peaking around 51 h post-adminstration.

### The origin of the ZnPc-EVs has a profound effect on the antitumor efficacy in murine models

**Fig. 6:**
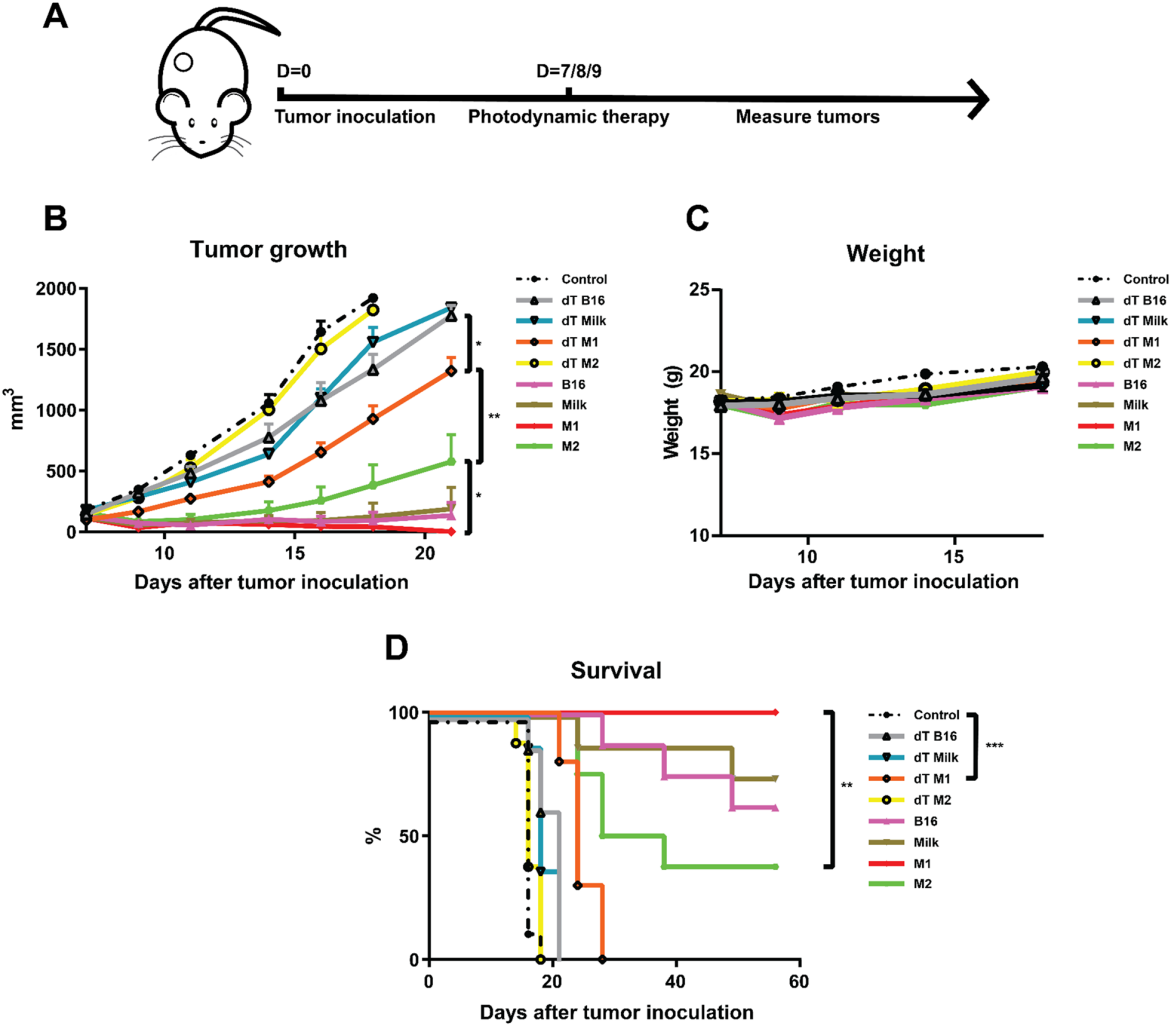
Photodynamic therapy in vivo. **a** Schematic representation of the protocol. C57BL/6 J mice were inoculated with 0.5 × 10^6^ MC38 cells in the right flank. At 7 days after inoculation, when tumors were established (~ 125 mm^3^), the mice were intravenously injected with the ZnPc-EVs in the tail vein. At 6, 24 and 48 h after administration, the tumors were illuminated with 690 nm light at 333 mW/cm^2^ for 100 J/cm^2^ per treatment, after which the tumor size was measured over time. **b** Tumor growth curves, **c** weight and **d** survival of the animals displayed over time. (*P<0.05; **P<0.01; ***P<0.0001; mean±SEM, n≥7)

**Fig. 7:**
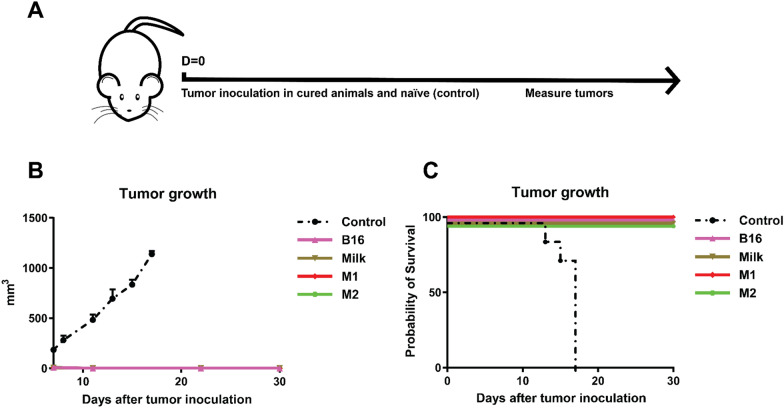
Tumor rechallenge in animals cured with the ZnPc-EVs. **a** Schematic representation of the protocol. Naïve C57BL/6 J mice (control) and C57BL/6 J mice cured after treatment with the ZnPc-EVs (Fig. 6) were inoculated with 0.5 × 10^6^ MC38 cells in the left flank, opposite to the previous inoculation site. **b** Tumor growth curves, and **c** survival of the animals are displayed over time. (mean±SEM, n≥3)

Expanding on the distribution data, we compared the antitumor efficacy of the ZnPc-EVs against MC38 tumors in C57BL/6 J mice. To this end, mice were subcutaneously inoculated with 0.5 × 10^6^ MC38 cells in the right flank. When the tumors became established (~ 125 mm^3^), the ZnPc-EVs were administered intravenously into the tail vein. Based on the distribution of the ZnPc-EVs, drug-to-light intervals of 6, 24 and 48 h were chosen, at which the tumors where illuminated with a 690 nm laser at 333 mW/cm^2^ for a total of 100 J/cm^2^ (Fig. 6a). In absence of light (dark toxicity, dT), the dT B16-ZnPc and dT Milk-ZnPc did not induce a notable tumor growth delay compared to control (Fig. 6b and Additional file 1: Fig. S4). However, the dT M1-ZnPc induced a significant tumor growth delay compared to all other dT EVs and to control animals, consistent with the in vitro cytotoxicity data (Fig. 3). The dT M2-EVs appeared to have no effect on MC38 tumor growth, with a near-identical growth curve compared to untreated (control) animals. For PDT in vivo with all ZnPc-EVs, the observed antitumor effect was enhanced compared to the dT M1-EVs (Fig. 6b). Again, the M2-EVs induced the least prominent tumor growth delay compared to PDT with all other ZnPc-EVs, with a statistically significant difference compared to the M1-EVs at day 21 after treatment (Fig. 6b). The B16-EVs and Milk-EVs induced a similarly strong tumor growth delay (Fig. 6b), in addition to comparable rates of complete responses in approximately 63–75% of animals lasting up to at least 60 days post inoculation (Fig. 6d). The M1-EVs showed the most efficient tumor growth delay (Fig. 6b) and complete responses in 100% of animals after treatment (Fig. 6d), which was significantly increased compared to the M2-EVs that only induced complete responses in 38% of animals. The treatment appeared to be well tolerated by all animals and did not induce significant changes in animal weight compared to control at day 18 after inoculation (Fig. 6c), suggesting that the treatment does not induce notable toxicity. Finally, the ability of the ZnPc-EVs to induce immunological memory was assessed by re-inoculating 0.5 × 10^6^ MC38 cells in the left (opposite) flank of only the animals that were tumor-free after the ZnPc-EV tumor treatment (Fig. 7a). Initially, all tumors became palpable at 5 days post inoculation, after which the tumors went into regression for all conditions except control (naïve) animals (Fig. 7b, c), suggesting the ability of treatment to induce immunolocial memory against MC38 tumors. Taken together, the in vivo results suggest that the origin of the extracellular vesicles can have a profound effect on the antitumor efficacy, both for the EVs administered in absence of light and for PDT in vivo, whereby EVs derived from M1-like macrophages are significantly more efficient versus EVs derived from M2-like macrophages. Moreover, the results suggest that all animals that respond by clearing their tumors after treatment develop immunological memory, indicating involvement of the immune system in the therapeutic response in spite of differences in therapeutic efficacy related to the initial tumor challenge.

## Discussion

The role of EVs in tumor development has been controversial, with EVs inducing both tumor progression and regression, depending on the immunological state and cell type from which the EVs were obtained. For instance, it was found that treatment with EVs isolated from mature dendritic cells inhibited tumor growth [25], whereas treatment with EVs derived from cancer cells promoted tumor growth, potentially through transfer of tumor-promoting miRNAs [26–28]. Moreover, an analysis of pre-therapy plasma of acute myeloid leukemia patients was shown to contain elevated levels of immunosuppressive EVs that interfered with cellular immunotherapy [35]. However, the cell type and immunological state from which the EVs were isolated remain unknown, complicating a comparison of the effects of the origin of the EVs in this study. In addition to this, the various EVs natively present in the tumor play a significant role in the interaction between cancer and immune cells, but originate from numerous cell types in different physiological and immunological states that vary over time as well as between cancer models. Therefore, the effects of therapeutic interventions using EVs are complex and difficult to interpret, often leading to paradoxical observations. In line with this, some studies suggested that tumor-derived EVs were able to polarize macrophages towards an M1-phenotype [36, 37], while others suggested polarization towards the M2- phenotype [38, 39], and still others suggested that EVs alone were insufficient to induce macrophage polarization [40]. In addition, there are multiple forces other than EVs at play in the tumor, further complicating an interpretation of the effects of the EV-treatment. For example, tumor-derived EVs induced macrophages with an M1-phenotype to produce inflammatory cytokines, but the tumor cells retained their ability to metastasize and escape immune surveillance, due to mechanisms that may or may not be related to EVs [41, 42]. The multitude of mechanisms by which cancer cells are able to escape immune surveillance and initiate metastases, further complicates the exact role of the therapeutic EVs in these processes.

In the present study, we have attempted to provide more clarity on the role of EVs in cancer treatment by directly comparing ZnPc-mediated photodynamic therapy with EVs derived from different cell types with different immunological states. Of these, the M1-derived EVs were the only ones to induce polarization of RAW 264.7 macrophages to an M1-like phenotype. Moreover, M1-EVs were toxic to MC38 cells at concentrations where the other ZnPc-EVs did not affect viability. All ZnPc-EVs were associated with both MC38 and D1DCs in mono-culture, but displayed a preference for MC38 over D1DCs in co-culture. Consistent with the results of the MTS assay, M1-ZnPc was toxic to MC38 cells but not to D1DCs in absence of light, both in mono- and in co-culture, whereas the other ZnPc-EVs failed to induce notable viability reduction in either cell line. PDT with all ZnPc-EVs induced near-complete cell death in both D1DCs and MC38 cells, showing the potential of ZnPc-PDT in an in vitro setting regardless of EV origin. PDT with all tested ZnPc-EVs was shown to induce strong exposure of CRT at levels higher than three cycles of freeze-thawing at − 20 °C (FT). Furthermore, incubation of D1DCs with PDT-treated cancer cells using the ZnPc-EVs, increased the dendritic cell maturation marker CD86 to levels comparable to incubation with direct TLR-ligand poly I:C. These results show the ability of PDT with the ZnPc-EVs to induce ICD in an in vitro setting. The distribution of the ZnPc-EVs was investigated in MC38 tumor-bearing mice, revealing an accumulation of the EVs in the tumors over time, with a peak at 51 h post-administration. Based on this, the PDT in vivo protocol was established, inducing complete and lasting responses for PDT with all ZnPc-EVs. However, only the M1-EVs induced complete responses in all animals and was significantly more efficient in terms of enhancing tumor growth delay and survival than the M2-EVs. In absence of light, only the M1-EVs induced a tumor growth delay without complete responses, consistent with the in vitro cytotoxicity data. These results indicate that the origin of the ZnPc-EVs can have a strong effect on the therapeutic outcome, whereby ZnPc-EVs derived from immune cells in a pro-inflammatory (M1-like) state outperform ZnPc-EVs derived from the same cell type in an immunosuppressive (M2-like) state. Moreover, the efficacy of the M1-ZnPc was also increased versus tumor cell- and milk-derived EVs, albeit to a lesser extent than compared to the M2-EVs. Our results show that the cell type from which the EVs are derived plays a role in the efficacy of EV-mediated PDT, but highlight the impact of the immunological state of the cell of origin. This observation should be taken into consideration when selecting EVs as drug carriers for PDT-based antitumor treatment.

To the best of our knowledge, we are the first to directly compare the effects of the cell type and its immunological state on the efficacy of EV-mediated PDT. The results corroborate literature reporting on the cancer cell death-inducing and pro-inflammatory capacities of M1-polarized macrophages. In line with this, EVs from M1-polarized macrophages were shown to enhance the efficacy of a B16F10 vaccination strategy, by inducing a pro-inflammatory state in the draining lymph node [43]. Moreover, it was shown that M1-derived exosome-like vesicles induced a slight tumor growth inhibition and induced M1 polarization in vivo, while enhancing anti-PD-L1 (aPD-L1) antibody therapy [44]. Our results are similar to a report showing increased expression of caspase-3 in breast cancer cells after incubation with M1-derived EVs [24]. In the same study, paclitaxel-containing M1-EVs induced significant tumor growth inhibition in 4T1 tumor-bearing mice. Importantly, they also found a significant antitumor efficacy of M1-EVs alone on tumor growth to a comparable degree as we observe in our study. Based on our observations and corresponding literature, we propose that the mechanism behind the efficacy of M1-ZnPc PDT consists of (I) direct tumor cell killing capacities of M1-ZnPc, (II) PDT-induced ICD that potentially initiates an antitumor immune response and (III) polarization of macrophages to an M1-phenotype and promotion of a proinflammatory environment in the tumor and/or draining lymph nodes. However, the components present in the M1-ZnPc that are responsible for the direct tumor-killing capacity remain unidentified and are a topic of investigation in future studies. Moreover, the mechanism behind the enhanced antitumor efficacy of M1-ZnPc versus M2-ZnPc has not been unraveled. A factor contributing to this may be the ability of the M1-ZnPc to induce higher concentrations of ZnPc inside of tumor cells. Although the tumor distribution of M2-ZnPc is comparable to that of M1-ZnPc, the M2-ZnPc may not be associated with the cells as efficiently as the M1-ZnPc, reducing the antitumor efficacy following PDT. In any case, additional investigation is required to provide details to the mechanisms driving the antitumor efficacy of the M1-ZnPc. In addition, future studies might capitalize on the antitumor efficacy and proinflammatory effects of PDT with the M1-ZnPc. A recent study reported that exosomal micro-RNA-16-5p decreases the expression of PD-L1 in gastric cancer, triggering a T cell immune response that slows tumor growth and slightly enhances aPD-L1 antibody treatment [45]. As M1-derived EVs were found to enhance immune checkpoint inhibition therapy, further enhancement of antitumor efficacy might be achieved by combining M1-ZnPc PDT with such therapies.

## Methods

### Cells

The murine melanoma cell line B16F10, Murine Colon 38 (MC38) carcinoma cell line and murine macrophage cell line RAW 264.7 were cultured in Iscove’s modified Dulbecco’s medium (IMDM; Lonza, Basel, Switzerland), supplemented with 2 mM glutamine (Gibco, Landsmeer, The Netherlands), 8% Fetal Bovine Serum (Greiner, Kremsmünster, Austria), 100 IU/mL penicillin Gibco, Landsmeer, The Netherlands), 100 IU/mL streptomycin (Gibco, Landsmeer, The Netherlands) and 25 μM β-mercaptoethanol (Sigma-Aldrich, Zwijndrecht, The Netherlands). For flow cytometry purposes, MC38 cells were lentivirally transduced with Cyan Fluorescent Protein (CFP) and sorted on a BD FACSARIA II based on CFP^+^ to obtain MC38CFP, as described [46]. D1 Dendritic cells (D1DCs) were cultured as described previously [47]. D1DC culture medium consisted of Iscove's Modified Dulbecco's Medium (Lonza, Basel, Switzerland) supplemented with 8% Fetal Calf Serum (Greiner, Austria), 100 IU/mL penicillin (Gibco, Landsmeer, The Netherlands), 2 mM glutamine (Gibco, Landsmeer, The Netherlands) and 25 μM β-mercaptoethanol (Sigma-Aldrich, Zwijndrecht, The Netherlands). RAW 264.7 were polarized to an M1 by incubation with 0.1 μg/mL LPS (Sigma-Aldrich, St. Louis, MO) and 0,1 µg/mL IFN-γ (Peprotech, Cranbury, NJ) or to M2-phenotype by incubation with 0,04 µg/mL IL-4 (Peprotech, Hamburg, Germany), respectively, for 24 h in 25 mL serum-free medium. All cells used were routinely tested for mycoplasma as well as MAP-tested before the start of experiments and maintained at 37 °C and 5% CO_2_ in an incubator (Panasonic,’s- Hertogenbosch, The Netherlands), unless indicated otherwise.

### Preparation of EVs

B16F10 cells, M1- or M2- polarized RAW 264.7 cells were grown to 80–90% confluency, washed three times with PBS and incubated with fresh serum-free media for further 48 h. The supernatant was collected and centrifuged at 300 × g for 10 min, followed by 2,000 × g for 20 min and another round of 10,000 × g for 30 min to remove cells and cellular debris. The resulting supernatant was subsequently incubated for 1 h at 4 °C with an EV-precipitation buffer (Cellsg, Cambridge, UK) and further centrifugated at 16,000 × *g* for 60 min to concentrate EVs. In parallel, defatted bovine milk was purchased from a local supermarket from 3 different brands and pooled together prior to EVs isolation using a previously described protocol [48]. Briefly, the milk was pre-warmed for 10 min at 37 °C and then mixed with acetic acid at a Milk/acetic acid ratio of 1/100 for 5 min at room temperature followed by centrifugation at 10,000 *g* for 10 min at 4 °C. The supernatant was sterile filtered with a 0.22-µm membrane and the supernatant was subsequently incubated for 1 h at 4 °C with the EV-precipitation buffer (Cellsg, Cambridge, UK). Precipitation of EVs was achieved by centrifugation at 16,000 × g for 60 min. To incorporate ZnPc, the resulting pellets of B16F10, polarized RAW 264.7 and Milk were resuspended in PBS and incubated with 9 mM ZnPc (Sigma-Aldrich, Zwijdrecht, The Netherlands) in DMSO in a ratio of 9:1 for 1 h at 4 °C. Samples were then purified using a size exclusion column (exospin, Cellgs, Cambridge, UK) and centrifugation at 50 × *g* for 1 min. Extraction of ZnPc-EVs was finally achieved by spinning 200 µL PBS at 50 × *g* for 1 min through the column.

### Cryo-electron microscopy

Cryo-electron microscopy (cryo-EM) was performed as described previously [29]. Briefly, samples were placed on a glow-discharged 300 mesh EM grid (Quantifoil R2/2) and vitrified using an EMGP (Leica, Germany) at room temperature and 100% humidity. Excess sample was removed by blotting for 1 s with a Whatman filter paper. The grids were plunged into liquid ethane (− 182 °C) and, following vitrification, the grid was stored in liquid nitrogen until further use. They were then mounted in a Gatan 626 cryo-holder for cryo-EM imaging using a Tecnai 12 electron microscope (FEI Company, the Netherlands) operated at 120 kV. The images were recorded on a 4 × 4 k Eagle camera (FEI Company, the Netherlands) at 18,000 × magnification (pixel size 1.2 nm) between 5 and 10 µm under focus.

### Fluorescence microscopy

The location of the ZnPc-EVs inside cells was investigated by seeding 3000 MC38 cells in an 8-chamber polystyrene vessel tissue culture-treated glass slides (Corning) and allowing them to attach overnight. This was followed by incubation for 6 and 24 h with the ZnPc-EVs with a concentration calibrated at 4 µM ZnPc. After this time, the cells were washed 3 times in PBS and fixed in PBS containing 1% formalin (J.T. Baker) at 4 °C for 20 min. The cells were then washed three times in PBS and stained with 0.25 µM DAPI (Sigma), after which the coverslips were mounted on the glass slides using Mowiol mounting medium (Sigma-Aldrich) with 2.5% w/v DABCO (Merck) and sealed with nail polish. The slides were then imaged on a Leica SP8 fluorescence microscope.

### Particle concentration, size distribution and protein content of EVs

To determine particle concentration and size distribution, samples were diluted in PBS and analyzed using a Nanosight® NS300 (Malvern), with a detection threshold of 3, camera level of 9 and automatic post-acquisition settings. Capillary electrophoresis was performed using the Wes® automated western blot testing (ProteinSimple) to detect EV-proteins as previously described (ref paper ZnPc1). Briefly, cell lysates and EV-samples were lysed in 20 mM HEPES, 0.5 mM phenylmethylsulfonyl fluoride (PMSF) and 50 mM phosphatase inhibitor ortho-vanadate in PBS and the total protein content was determined with a MicroBCA protein assay kit (Thermo Fisher). 0.8 µg/µL of lysed proteins were mixed with the provided SDS/DTT mix and boiled at 95 °C. In parallel, the primary antibodies (rabbit) anti-Grp94 (Sigma, 1:10), anti-Flotillin-1 (Cell signal, 1:10), anti-HSP70 (Cell signal, 1:100), anti-α6-integrin (Cell signal 1:10) and anti-β-actin (BioLegend, 1:50) were diluted in the provided antibody diluent. A prefilled microwell plate was then loaded with the resulting proteins, primary antibodies, blocking buffer, luminol/peroxidase, HRP streptavidin, and secondary anti-rabbit antibody provided by the manufacturer (anti rabbit detection module, protein simple). The plate was then centrifuged at 300 × g for 5 min and electrophoretic separation was performed using 25-capillary cartridges for 12–230 kDa protein separation (SM-W004). Chemiluminescent bands were digitally generated and analyzed using the Compass software (ProteinSimple).

### Polarization of macrophages by the EVs

RAW 264.7 cells were seeded in 24-well plates at a density 5 × 10^5^ cells/well in a 12-well plate. At 80–90% confluency, the cells were incubated with the ZnPc-EVs for 24 h in serum-free medium. As controls, cells were incubated with LPS/ IFN-γ (both at 0,1 µg/mL, as described above) for M1 polarization or 0,04 µg/mL IL-4 (Peprotech, lot# 021,749 J2418) for M2 polarization. Cells were then collected, washed in PBS and reconstituted in Fluorescence-Activated Cell Sorting (FACS) buffer (PBS with 0.5% Bovine Serum Albumin (BSA) and 0.02% sodium azide). The cells were then stained with Anti-mouse CD163- PerCP (eBioscience, San Diego, CA, USA) or anti-mouse CD68-FITC (eBioscience, San Diego, CA, USA) and analyzed by flow cytometry on an LSR II (BD Biosciences, San Jose, CA, USA).

### In vitro* cytoxicity of EVs*

The cytotoxicity of the ZnPc-EVs was evaluated by the 3-(4,5-dimethylthiazol-2-yl)-5-(3-carboxymethoxyphenyl)-2-(4-sulfophenyl)-2H-tetrazolium (MTS) assay (Promega, Leiden, The Netherlands). MC38 cells were seeded in 96-well plates to a density of 4 × 10^64^, after which the medium was replaced with 100 μL of increasing concentrations of ZnPc-EVs in culture medium without serum and incubated for an additional 24, 48 and 72 h. The cell viability was measure using the MTS reagent according to the manufacturers’ protocol using at least 3 replicates for each experimental condition.

### Cellular association of ZnPc-EVs

To determine the association of the ZnPc-EVs in mono-culture, 5 × 10^4^ MC38 cells or 1 × 10^5^ D1DCs were seeded in 24-well plates (Corning, Glendale, CA, USA) in their respective culture media and incubated overnight at 37 °C and 5% CO_2_. Cells were then incubated with the ZnPc-EVs with a ZnPc concentration calibrated at 4 µM for a specified time. Following incubation, the cells were washed 3 times with PBS and fixed in PBS containing 1% formalin (J.T. Baker, Landsmeer, The Netherlands) at 4 °C for 15 min. The fixative was washed three times with PBS and the cells were reconstituted in FACS buffer. Samples were then stained with 0.5 µM of viability marker 4′,6-diamidino-2-phenylindole (DAPI) (Sigma-Aldrich, Zwijndrecht, The Netherlands), and association was determined by measuring the fluorescence of the photosensitizer by flow cytometry on a Cytek Aurora 3-Laser flow cytometer (Cytek, Fremont, CA, USA). To determine the association of the ZnPc-EVs in co-culture, 3 × 10^4^ MC38CFP cells were seeded in 24-well plates and incubated overnight at 37 °C and 5% CO_2_. The next morning, the cells were counted and an equal amount of D1DCs was added. Cells were then incubated with the ZnPc-EVs with an ZnPc concentration calibrated at 4 µM for 4 h, washed and reconstituted in FACS buffer. To distinguish cancer cells (MC38CFP) from D1DCs, the cells were stained with anti-CD11c-PE (Clone HL3, BD Biosciences, New Jersey, USA), followed by analysis on a Cytek Aurora 3-Laser flow cytometer.

### *PDT *in vitro* cytotoxicity*

For PDT in mono-culture, 4 × 10^4^ MC38, or 1 × 10^5^ D1DCs were seeded in 24-well plates in their respective culture media and incubated overnight at 37 °C and 5% CO_2_. Cells were then incubated with ZnPc-EVs with a ZnPc concentration calibrated at 4 µM for 4 h. Cells were then washed 3 times with PBS and supplied with 500 µL fresh medium. Illumination was performed at a light intensity (fluence rate) of 200 mW/cm^2^ for a total light dose (fluence) of 20 J/cm^2^ using a 690 nm LED Laser (CNI lasers, Changchun, China). Cells were then incubated for 18 h, collected in FACS buffer, stained with 0.5 µM DAPI in FACS buffer and analyzed by flow cytometry on a Cytek Aurora 3-Laser flow cytometer. As a positive control, cells were subjected to three freeze/thaw cycles at −20 °C (FT) before staining and analysis by flow cytometry on a Cytek Aurora 3-Laser flow cytometer (Cytek, Fremont, USA). For PDT in co-culture, 3 × 10^4^ MC38CFP cells were seeded in 24-well plates and incubated overnight at 37 °C and 5% CO_2_. The next morning, the cells were counted and an equal amount of D1DCs was added. Cells were then incubated with the ZnPc-EVs with a ZnPc concentration calibrated at 4 µM for 4 h, treated with PDT or FT, and analyzed as described.

### *Detection of DAMPs after PDT *in vitro

To measure the exposure of calreticulin (CRT) and release of HMGB-1, 4 × 10^4^ MC38 cells were seeded in 24-well plates in culture medium and incubated overnight at 37 °C and 5% CO_2_. Cells were then incubated with ZnPc-EVs with a ZnPc concentration calibrated at 4 µM for 4 h and treated with PDT or FT as described. Cells were then incubated for 18 h, after which the supernatant was collected and frozen at − 20 °C until further processing to measure HMGB-1 release by enzyme-linked immunosorbent assay (ELISA). After collection of the supernatant, the remaining cells were washed 3 times in PBS, collected in FACS buffer, stained with 0.5 µM DAPI and the antibody anti-calreticulin-PE (Clone EPR3924, Abcam, Cambridge, UK) in FACS buffer before analysis by flow cytometry on a Cytek Aurora 3-Laser flow cytometer. Frozen supernatants were thawed after which accidental cells were removed by centrifugation at 300 × g for 5 min. Supernatants were incubated in 96-well NUNC Maxisorp plates (Thermofisher, Landsmeer, The Netherlands) pre-treated overnight at 4 °C with 50 µL of a 5 µg/mL rabbit-anti-HMG-1/HMGB-1 antibody (Novus Biologicals, Centennial, CO, USA) in coating buffer (0.05 M Carbonate-Bicarbonate, pH 9.6). Plates were then washed 3 times in washing buffer (PBS with 0.05% Tween 20, pH 8.0) and incubated with blocking buffer (PBS with 0.05% Tween and 1% BSA, pH 8.0) for 60 min at 37 °C. The plates were then washed 3 times in washing buffer and incubated with 150 µL of the samples for 120 min at 37 °C. The plates were then washed 3 times in washing buffer and incubated with 4 µg/mL mouse-anti human HMG-1/HMGB-1-Biotin (Clone 19N12A1, Novus Biologicals, Centennial, CO, USA) in blocking buffer at room temperature for 60 min. The plates were again washed 3 times in washing buffer and incubated with Streptavidin-poly-HRP (Thermofisher, Landsmeer, The Netherlands) in blocking buffer for 60 min at room temperature. Plates were then washed 5 times in washing buffer, dried briefly and incubated with HRP-substrate Temozolomide until a change of color was clearly visible. The reaction was stopped by addition of 0.18 M H_2_SO_4_ in deionized water, after which the absorption was measured at 490 nm using a Bio-Rad iMark microplate absorbance reader (Bio-Rad Laboratories, Veenendaal, The Netherlands).

### Maturation of D1DCs after incubation with PDT-treated tumor cells

The immunostimulatory effects of PDT were investigated by seeding 5 × 10^5^ MC38CFP cells in 24-well plates and 10^4^ D1DCs in 96-well plates (Corning, Glendale, CA, USA). After overnight incubation at 37 °C and 5% CO_2_, the cancer cells were incubated with the ZnPc-EVs with a ZnPc concentration calibrated at 4 µM for 4 h and treated with PDT or FT as described. The (dying) treated tumor cells were then added to the D1DCs at a ratio of 20: 1 (tumor cell: D1DC) and incubated for 24 h at 37 °C and 5% CO_2_. The cells were then collected, stained with 0.5 µM DAPI (Sigma-Aldrich, Zwijndrecht, The Netherlands), anti-CD11c-PE (Clone HL3, BD Biosciences, New Jersey, USA) and CD86-FITC (clone GL1, Thermofisher, Landsmeer, The Netherlands), and finally, analyzed by flow cytometry on a Cytek Aurora 3-Laser flow cytometer. Controls consisted of D1DCs in mono-culture (-), poly I:C at indicated concentration, ZnPc-EVs directly incubated with D1DCs, or ZnPc-EVs incubated with tumor cells added to D1DCs without PDT.

### Animals

Female C57BL/6 J mice were obtained from ENVIGO (Horst, the Netherlands) and C57BL/6-albino mice were bred in the breeding facility of the Leiden University Medical Center (LUMC, Leiden, The Netherlands). All animals were housed under specified pathogen-free conditions in the animal facility of the LUMC. The animal experiments were conducted in accordance with the Code of Practice of the Dutch Animal Ethical Commission (animal permit: AVD1160020198405, approved 19 November 2019).

### *Distribution of ZnPc-EVs *in vivo

For imaging in vivo, C57BL/6-albino mice were inoculated subcutaneously with 5 × 10^5^ MC38 in 200 µL PBS on right flank. Once the tumors had reached an average volume of approximately 125 mm^3^, the mice were randomly divided into groups and the ZnPc-EVs were administered intravenously into the tail vein at 400 µM in 100 µL PBS. The fluorescence of ZnPc was then measured in the tumors over time, by fluorescence spectrometry imaging using the IVIS Spectrum (Perkinelmer, Waltham, MA, USA) under isoflurane anesthesia. Relevant areas were shaved right before measurement to minimize interference of the fluorescent signal. Measurements were performed at automatic exposure times at position C using filter settings relevant for ZnPc.

### PDT using ZnPc-EVs in vivo and rechallenge in animals with complete responses

For PDT in vivo, C57BL/6 J mice were inoculated subcutaneously with 5 × 10^5^ MC38 in 200 µL PBS in the right flank. Once the tumors had reached an average volume of approximately 125 mm^3^, the mice were randomly divided into groups and treated with PDT. The ZnPc-EVs were administered intravenously into the tail vein at 400 µM in 100 µL PBS. At a drug-to-light interval (DLI) of 6, 24 and 48 h, the skin surrounding the tumor area was shaved and tumors were illuminated with 690 nm light under isoflurane anesthesia at a fluence rate of 333 mW/cm^2^ over 300 s for a fluence of 100 J/cm^2^. Mouse conditions were checked regularly, and tumor sizes were measured three-weekly using a caliper until the end of the experiment. Animals that displayed complete responses were rechallenged with 5 × 10^5^ MC38 in 200 µL PBS in the left (opposite) flank. Mouse conditions were checked regularly, and tumor sizes were measured three-weekly using a caliper until the end of the experiment.

### Statistics

Graph Pad Prism software version 9 was used for statistical analysis, FlowJo was used for flow cytometry data and Living Image was used for processing biodistribution in vivo data obtained with the IVIS Spectrum. Data were analyzed as indicated for individual experiments.

## Supplementary Information

**Additional file 1: Figure S1.** Fluorescence microscopy and cryo-TEM images. **Figure S2.** Association of ZnPc-EVs with dendritic cells and cancer cells. **Figure S3.** ZnPc-EV-mediated PDT moderately induces HMGB-1 release. **Figure S4.** Tumor growth curves of photodynamic therapy in vivo.

## Data Availability

The data can be made available online if desired.
