# Supplementary figures and images for: M1-derived extracellular vesicles enhance photodynamic therapy and promote immunological memory in preclinical models of colon cancer
Source: J Nanobiotechnology. 2022 Jun 3;20:252. doi: 10.1186/s12951-022-01448-z (PMC9164362; doi:10.1186/s12951-022-01448-z)

**Additional file 1**


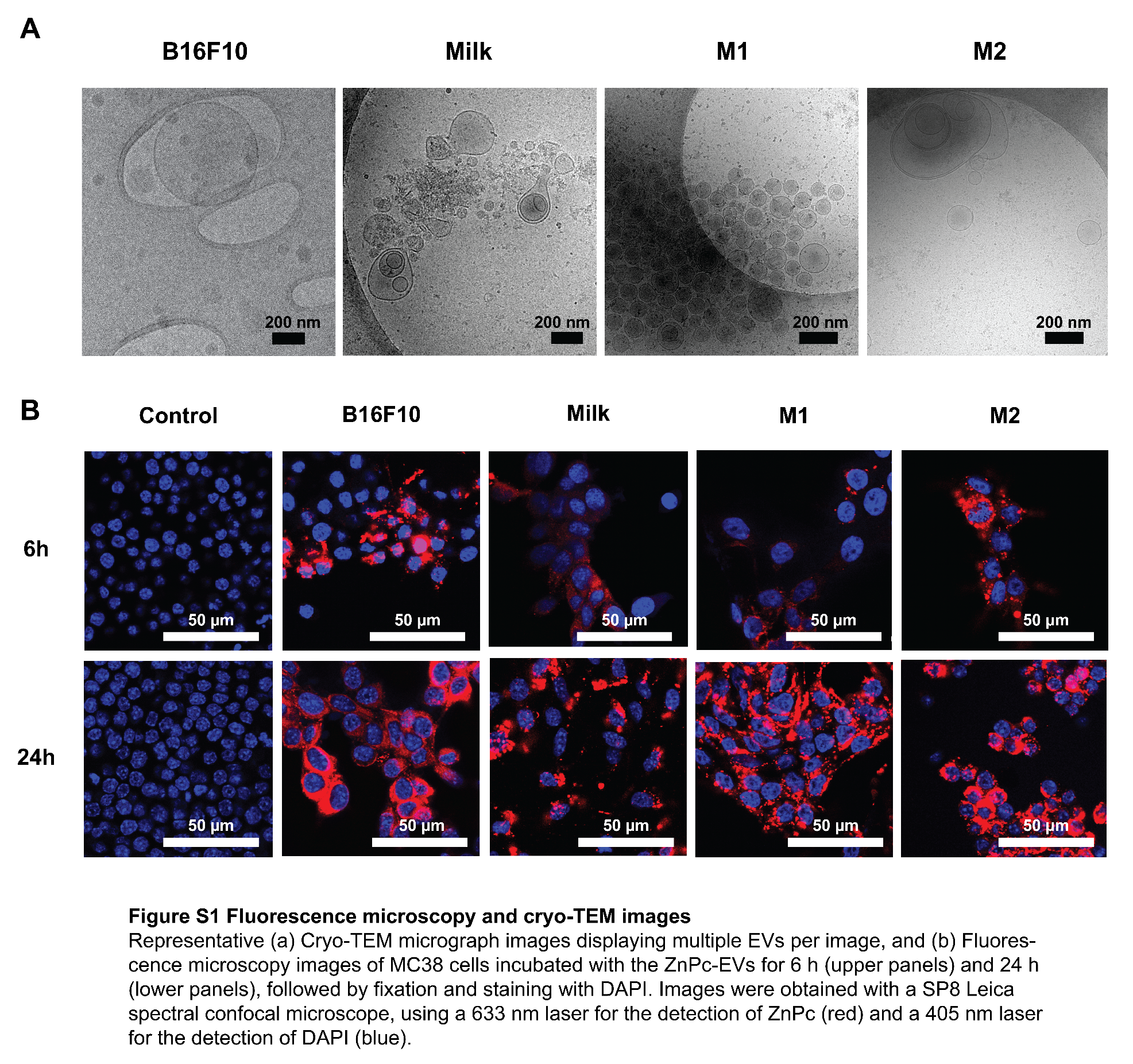


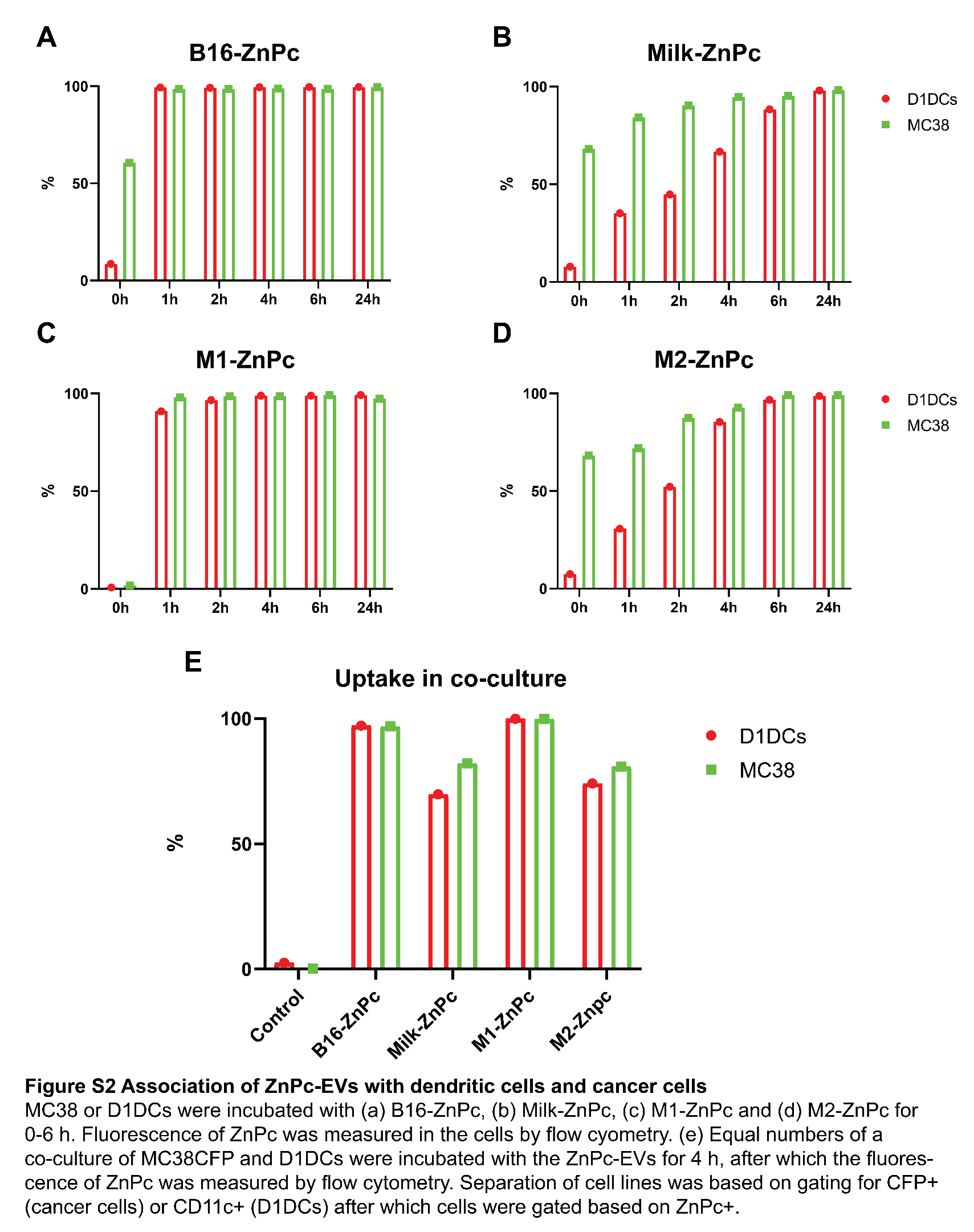


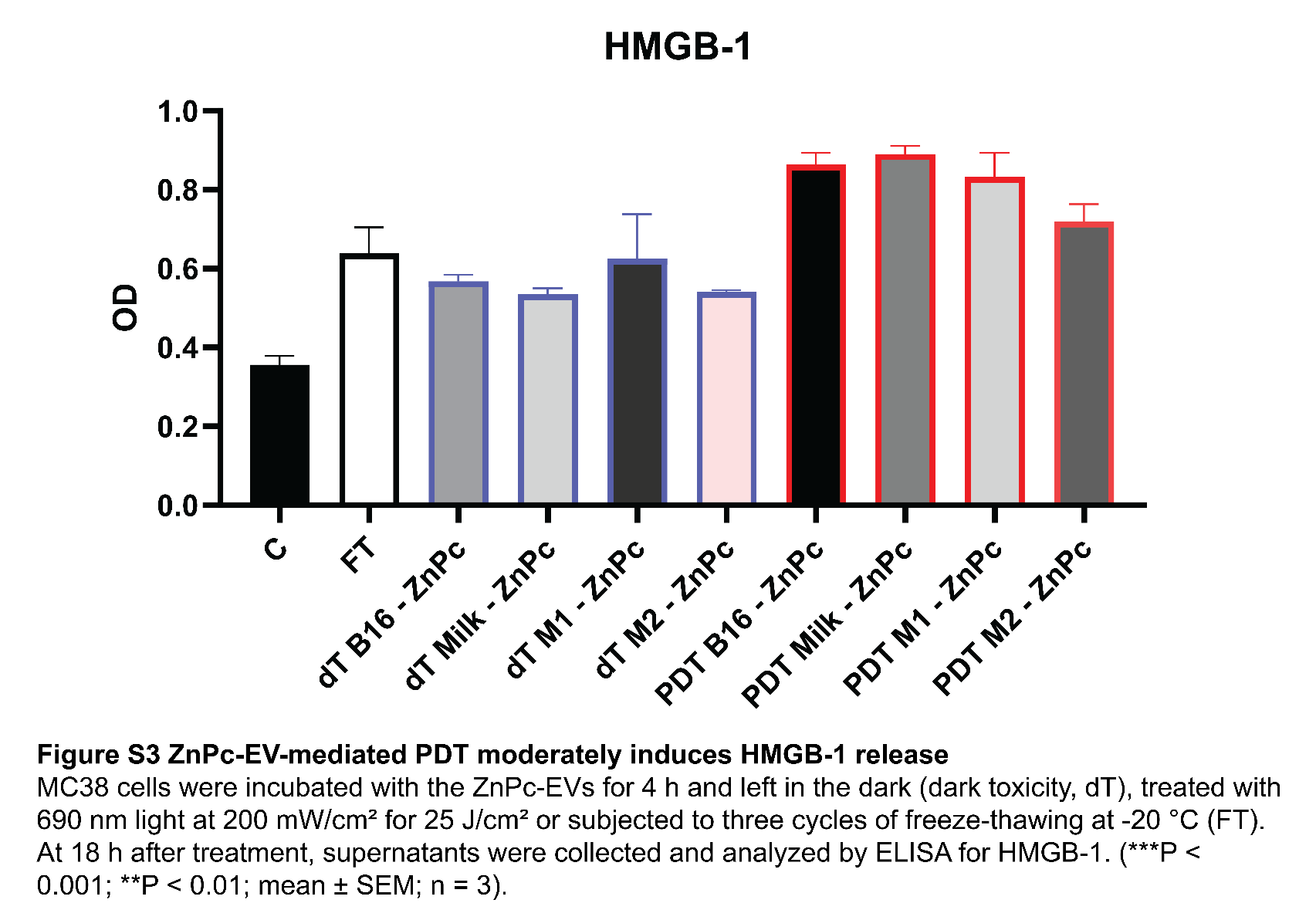


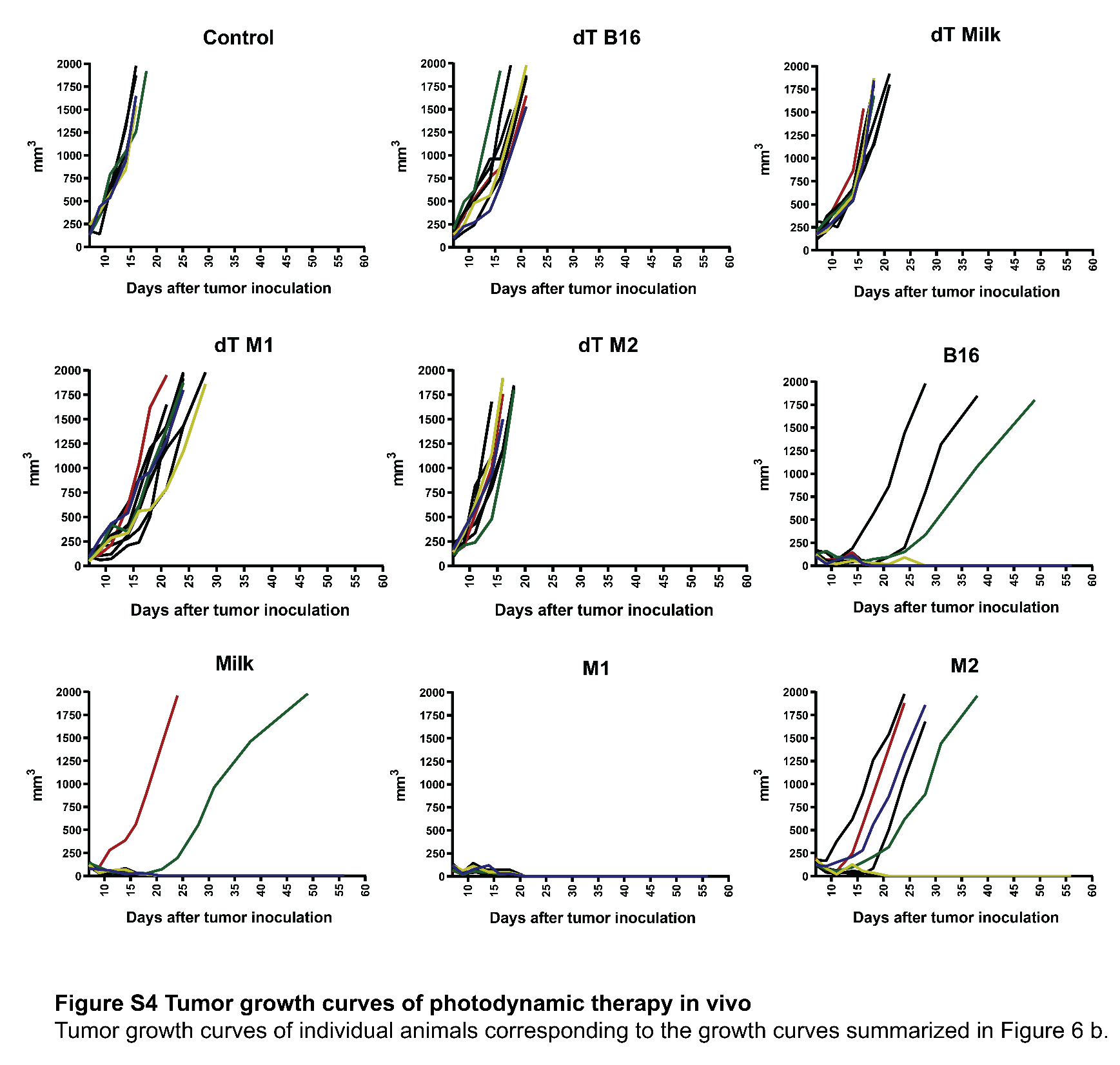

Supplement: Supplementary file 1 — Additional file 1: Figure S1. Fluorescence microscopy and cryo-TEM images. Figure S2. Association of ZnPc-EVs with dendritic cells and cancer cells. Figure S3. ZnPc-EV-mediated PDT moderately induces HMGB-1 release. Figure S4. Tumor growth curves of photodynamic therapy in vivo. [file 12951_2022_1448_MOESM1_ESM.docx]
